# Multiple Layers of Regulation on Leaf Senescence: New Advances and Perspectives

**DOI:** 10.3389/fpls.2021.788996

**Published:** 2021-12-06

**Authors:** Yue-Mei Zhang, Pengru Guo, Xinli Xia, Hongwei Guo, Zhonghai Li

**Affiliations:** ^1^National Engineering Laboratory for Tree Breeding, College of Biological Sciences and Technology, Beijing Forestry University, Beijing, China; ^2^Key Laboratory of Molecular Design for Plant Cell Factory of Guangdong Higher Education Institutes, Department of Biology, Southern University of Science and Technology, Shenzhen, China

**Keywords:** leaf senescence, senescence-associated genes, multi-omics, gene regulatory network, alternative splicing

## Abstract

Leaf senescence is the last stage of leaf development and is an orderly biological process accompanied by degradation of macromolecules and nutrient recycling, which contributes to plant fitness. Forward genetic mutant screening and reverse genetic studies of senescence-associated genes (SAGs) have revealed that leaf senescence is a genetically regulated process, and the initiation and progression of leaf senescence are influenced by an array of internal and external factors. Recently, multi-omics techniques have revealed that leaf senescence is subjected to multiple layers of regulation, including chromatin, transcriptional and post-transcriptional, as well as translational and post-translational levels. Although impressive progress has been made in plant senescence research, especially the identification and functional analysis of a large number of SAGs in crop plants, we still have not unraveled the mystery of plant senescence, and there are some urgent scientific questions in this field, such as when plant senescence is initiated and how senescence signals are transmitted. This paper reviews recent advances in the multiple layers of regulation on leaf senescence, especially in post-transcriptional regulation such as alternative splicing.

## Introduction

Plant leaves are the main organ for photosynthesis, converting light energy into chemical energy stored in carbohydrate molecules, which is the main source of energy for all organisms on earth. Senescence is the final stage of leaf development process, which is a slow and complex biological process including the initiation, progression, and terminal phases (Guo and Gan, 2005; Lim et al., 2007). The degradation of chlorophyll and chloroplasts occurs in the later phase of leaf senescence, accompanied by the degradation of macromolecules such as proteins, lipids, and nucleic acids. In annual plants, the nutrients released from senescent leaves are transferred to actively growing young leaves and seeds to increased reproductive success. In perennial plants such as deciduous trees, the nitrogen from leaf proteins is relocated to form bark storage proteins (BSP) in phloem tissues, and then remobilized and reutilized for spring shoot growth. Therefore, the timing of leaf senescence plays an important role in ensuring nutrient recycling, adaptation to the environment, and reproduction in plants. A number of studies in crops such as wheat and rice revealed that alteration of leaf senescence process could significantly affect the yield and quality of crops. Extended lifespan of leaves in apple trees greatly improved fruit quality in apple trees (Han et al., 2020; Hu et al., 2020), and increased fruit yield and sugar content in tomato (*Solanum lycopersicon*) (Lira et al., 2017; Ma et al., 2018). Moreover, delayed leaf senescence conferred enhance drought resistance in tobacco or cassava (Zhang et al., 2010). Therefore, an in-depth understanding of the regulatory mechanisms of leaf senescence is of great importance.

Leaf senescence is not a passive and disorderly process, but a highly programmed degenerative process (Guo and Gan, 2005). The initiation and progression of leaf senescence are influenced by numerous endogenous developmental signals and external environmental factors. Leaf age is the most important endogenous cue that determines the initiation of leaf senescence. However, the nature of age and how age information is perceived remains a mystery (Jing et al., 2002). Plant hormones such as ethylene, jasmonic acid (JA), salicylic acid (SA), abscisic acid (ABA), brassinosteroid (BR), and strigolactone (SL) promote leaf senescence and are extensively involved in response to various abiotic and biotic stresses, whereas auxin, cytokinins (CKs), and gibberellins (GAs) delay leaf senescence (Lim et al., 2007; Miao and Zentgraf, 2007; Li et al., 2013; Zhang et al., 2013; Yamada and Umehara, 2015; Kim et al., 2020). Hormone signaling pathways often mediate or influence development and environmental responses to regulate leaf senescence (Lim et al., 2007). Interestingly, changes in the circadian rhythm of plants also impact leaf senescence, but the causal relationship between them needs to be further explored (Song et al., 2018). In addition to being regulated by plant age or phytohormones, leaf senescence can also be caused by numerous environmental stresses such as darkness, nutrient deficiency, drought stress, and pathogen infection (Lim et al., 2007; Chen et al., 2013; Woo et al., 2019; Li et al., 2020b). There is much information about age- or abiotic stress-induced leaf senescence, whereas little is known about the molecular basis of biotic stress-triggered senescence. Recently, it was found that the secretory effector protein PevD1 (Protein elicitor from *Verticillium. dahliae* 1) plays an important role in the *V. dahliae*–induced senescence process. PevD1 interacts with ORESARA1 (ORE1), one core transcription factor regulating plant senescence (Lim et al., 2007), and attenuates the NLA-mediated degradation of ORE1, thereby enhancing ethylene biosynthesis by directly binding the promoter of 1-AMINOCYCLOPROPANE-1-CARBOXYLIC ACID (ACC) SYNTHASE 6 (ACS6) (Zhang Y. et al., 2021). This research provides a mechanism for previous observations that ethylene contributes to *V. dahliae*–induced premature leaf senescence.

## Multiple-Layers of Regulation on Leaf Senescence

In the past few decades, remarkable progress has been made in leaf senescence research, and time-evolving genetic networks have been established through genetics and multi-omics strategies, allowing us to gain a deeper understanding of this important biological process (Kim H. J. et al., 2018).

**FIGURE 1:**
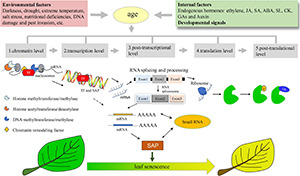
Multiple Layers of Regulation on Plant Leaf Senescence. Plant leaf senescence is finely regulated by endogenous developmental signals, and external environmental cues in an age-dependent manner. The multiple layers of regulation on leaf senescence includes epigenetic regulation at the chromatin level involved in histone proteases, DNA methylation modifying enzymes and chromatin remodeling factors, the master transcription factors involved in transcription regulation such as WRKYs and NACs, also includes miRNA and alternative splicing involved in the post-transcriptional level, and translation initiation and elongation factors involved in the regulation of translation level, as well as post-translational ubiquitination and phosphorylation. SAP, senescence-associated protein; Ub, ubiquitin; TF, transcription factors.

Here, we reviewed the recent advances in the molecular regulation of leaf senescence, including chromatin level, transcription level, as well as post-transcriptional, translational, and post-translational level (Figure 1). We also summarized the key players involved in the multilevel regulation of leaf senescence (Table 1).

**TABLE 1:** List of the key genes involved in multiple layers of regulation on leaf senescence.

**Gene**	**Species**	**Effects**	**Regulation**	**References**
AtHD1	*Arabidopsis thaliana*	Delay	Chromatin level	Pandey et al., 2002
AtSRT1	*Arabidopsis thaliana*	Delay	Chromatin level	Liu et al., 2017
BRAHMA	*Arabidopsis thaliana*	Delay	Chromatin level	Efroni et al., 2013
DRD1/DDM1	*Arabidopsis thaliana*	Promote	Chromatin level	Cho et al., 2016
HAC1	*Arabidopsis thaliana*	Promote	Chromatin level	Hinckley et al., 2019
HDA9	*Arabidopsis thaliana*	Delay	Chromatin level	Zheng et al., 2016
HDA15	*Arabidopsis thaliana*	Delay	Chromatin level	Shen et al., 2019
HDA19	*Arabidopsis thaliana*	Delay	Chromatin level	Ueda et al., 2018
HD2C	*Arabidopsis thaliana*	Delay	Chromatin level	Buszewicz et al., 2016
JMJ16	*Arabidopsis thaliana*	Promote	Chromatin level	Liu et al., 2019
SUVH2	*Arabidopsis thaliana*	Delay	Chromatin level	Ay et al., 2009
REF6	*Arabidopsis thaliana*	Promote	Chromatin level	Wang et al., 2019
WRKY6	*Arabidopsis thaliana*	Promote	Transcription level	Robatzek and Somssich, 2001
WRKY22	*Arabidopsis thaliana*	Promote	Transcription level	Zhou et al., 2011
WRKY42	*Arabidopsis thaliana*	Promote	Transcription level	Niu et al., 2020
WRKY45	*Arabidopsis thaliana*	Promote	Transcription level	Chen et al., 2017
WRKY46	*Arabidopsis thaliana*	Promote	Transcription level	Zhang D. et al., 2021
WRKY53	*Arabidopsis thaliana*	Promote	Transcription level	Zentgraf et al., 2010
WRKY54/WRKY70	*Arabidopsis thaliana*	Delay	Transcription level	Besseau et al., 2012
WRKY55	*Arabidopsis thaliana*	Promote	Transcription level	Wang et al., 2020
WRKY57	*Arabidopsis thaliana*	*Delay*	*Transcription level*	Jiang et al., 2014
WRKY75	*Arabidopsis thaliana*	Promote	Transcription level	Guo P. et al., 2017
GhWRKY42	*Gossypium hirsutum*	Promote	Transcription level	Gu et al., 2018
GhWRKY91	*Gossypium hirsutum*	Delay	Transcription level	Gu et al., 2019
CpWRKY71	*Chimonanthus praecox*	Promote	Transcription level	Huang et al., 2019
OsWRKY42	*Oryza sativa*	Promote	Transcription level	Han et al., 2014
OsWRKY93	*Oryza sativa*	Promote	Transcription level	Li Y. et al., 2021
BnaWSR1	*Brassica napus*	Promote	Transcription level	Cui et al., 2020
BnaWGR1	*Brassica napus*	Promote	Transcription level	Yang et al., 2018
BrWRKY6	*Brassica rapa var. parachinensis*	Promote	Transcription level	Fan et al., 2018
AtNAP	*Arabidopsis thaliana*	Promote	Transcription level	Guo and Gan, 2006
AtNAC3	*Arabidopsis thaliana*	Promote	Transcription level	Hickman et al., 2013
ATAF1	*Arabidopsis thaliana*	Promote	Transcription level	Garapati et al., 2015
ANAC016	*Arabidopsis thaliana*	Promote	Transcription level	Kim et al., 2013
ANAC017, VNI1, and ANAC090	*Arabidopsis thaliana*	Delay	Transcription level	Woo et al., 2016
ANAC019	*Arabidopsis thaliana*	Promote	Transcription level	Lee et al., 2015
ANAC032	*Arabidopsis thaliana*	Promote	Transcription level	Mahmood et al., 2016
ANAC046	*Arabidopsis thaliana*	*Promote*	*Transcription level*	Oda-Yamamizo et al., 2016
ANAC072	*Arabidopsis thaliana*	*Promote*	*Transcription level*	Li S. et al., 2016
NAC075	*Arabidopsis thaliana*	Delay	Transcription level	Kan et al., 2021
ANAC102	*Arabidopsis thaliana*	Promote	Transcription level	Nakashima et al., 2012
JUB1	*Arabidopsis thaliana*	Delay	Transcription level	Wu et al., 2012
NTL9	*Arabidopsis thaliana*	*Promote*	*Transcription level*	Yoon et al., 2008
ORE1	*Arabidopsis thaliana*	Promote	Transcription level	Kim et al., 2009
ORS1	*Arabidopsis thaliana*	Promote	Transcription level	Balazadeh et al., 2011
PIF4 and PIF5	*Arabidopsis thaliana*	Promote	Transcription level	Sakuraba et al., 2014; Song et al., 2014
VNI2	*Arabidopsis thaliana*	Delay	Transcription level	Yang et al., 2011
BnaNAC87	*Brassica napus*	Promote	Transcription level	Yan et al., 2018
lbNAC1	*Ipomoea batatas*	Promote	Transcription level	Chen et al., 2016
MpSNAC67	*Musa x paradisiaca*	Promote	Transcription level	Tak et al., 2018
MlNAC5	*Miscanthus lutarioriparius*	Promote	Transcription level	Yang et al., 2015
NtNAC080	*Nicotiana tabacum*	Promote	Transcription level	Li et al., 2018
NAM-B1	*Triticum turgidum*	Promote	Transcription level	Uauy et al., 2006
TaNAC-S	*Triticum turgidum*	Delay	Transcription level	Zhao et al., 2015
ONAC011	*Oryza sativa*	Promote	Transcription level	El Mannai et al., 2017
ONAC096	*Oryza sativa*	Delay	Transcription level	Kang et al., 2019
ONAC106	*Oryza sativa*	Delay	Transcription level	Sakuraba et al., 2015
OsNAC2	*Oryza sativa*	Promote	Transcription level	Mao et al., 2017
OsNAP	*Oryza sativa*	Promote	Transcription level	Liang et al., 2014
OsDOS	*Oryza sativa*	Delay	Transcription level	Kong et al., 2006
SlNAP1 and SlNAP2,	*Solanum lycopersicon*	Promote	Transcription level	Ma et al., 2018
SlORE1S06, SlORE1S03, and SlORE1S02	*Solanum lycopersicon*	Promote	Transcription level	Lira et al., 2017
SiNAC1	*Setaria italica*	Promote	Transcription level	Ren et al., 2018
GmNAC065 and GmNAC085	*Glycine max*	Promote	Transcription level	Melo et al., 2018
GmNAC81	*Glycine max*	Promote	Transcription level	Pimenta et al., 2016
GhNAC12	*Gossypium hirsutum*	Promote	Transcription level	Zhao et al., 2016
AIF2	*Arabidopsis thaliana*	Delay	Transcription level	Kim et al., 2020
AP2	*Arabidopsis thaliana*	Delay	Transcription level	Balanza et al., 2018
bHLH03, bHLH13, bHLH14, and bHLH17	*Arabidopsis thaliana*	Delay	Transcription level	Qi et al., 2015
CRF1, CRF2, CRF3, CRF5, and CRF6	*Arabidopsis thaliana*	Promote	Transcription level	Raines et al., 2016
CBF2	*Arabidopsis thaliana*	Delay	Transcription level	Sharabi-Schwager et al., 2010
DEAR1	*Arabidopsis thaliana*	Promote	Transcription level	Tsutsui et al., 2009
FYF	*Arabidopsis thaliana*	Delay	Transcription level	Chen et al., 2011
IAA17	*Arabidopsis thaliana*	Promote	Transcription level	Shi et al., 2015
KHZ1 and KHZ2	*Arabidopsis thaliana*	Promote	Transcription level	Yan et al., 2017
MYC2, MYC3, and MYC4	*Arabidopsis thaliana*	Promote	Transcription level	Qi et al., 2015
MYC5	*Arabidopsis thaliana*	Promote	Transcription level	Song et al., 2017
JAZ7	*Arabidopsis thaliana*	Promote	Transcription level	Yu et al., 2016
MdbHLH3	*Malusdomestica*	Promote	Transcription level	Hu et al., 2020
AtMYBL	*Arabidopsis thaliana*	Promote	Transcription level	Zhang et al., 2011
MYB2	*Arabidopsis thaliana*	Promote	Transcription level	Buchanan-Wollaston et al., 2005
MYBR1	*Arabidopsis thaliana*	Delay	Transcription level	Jaradat et al., 2013
MYBH	*Arabidopsis thaliana*	Promote	Transcription level	Huang et al., 2015
ORE15	*Arabidopsis thaliana*	Delay	Transcription level	Kim J. H. et al., 2018
OsMYC2	*Oryza sativa*	Promote	Transcription level	Uji et al., 2017
OsMYB102	*Oryza sativa*	Delay	Transcription level	Piao et al., 2019
Rap2.4f	*Arabidopsis thaliana*	Promote	Transcription level	Xu et al., 2010
REVOLUTA	*Arabidopsis thaliana*	Promote	Transcription level	Xie Y. et al., 2014
RAV1	*Arabidopsis thaliana*	Promote	Transcription level	Woo et al., 2010
ScMYB2S1	*Sugarcane*	Promote	Transcription level	Guo X. et al., 2017
SlERF36	*Solanum lycopersicon*	Promote	Transcription level	Upadhyay et al., 2013
SUB1A	*Oryza sativa*	Delay	Transcription level	Fukao et al., 2012
SlFYFL	*Solanum lycopersicon*	Delay	Transcription level	Xie Q. et al., 2014
SlMBP11	*Solanum lycopersicon*	Delay	Transcription level	Guo X. et al., 2017
TCP2, TCP4, and TOP10	*Arabidopsis thaliana*	Promote	Transcription level	Schommer et al., 2008
CFM4	*Arabidopsis thaliana*	Promote	Post-transcriptional level	Lee et al., 2014
ERF4	*Arabidopsis thaliana*	Promote	Post-transcriptional level	Koyama et al., 2013; Riester et al., 2019
miR156	*Arabidopsis thaliana*	Delay	Post-transcriptional level	Wang, 2014
miR164	*Arabidopsis thaliana*	Delay	Post-transcriptional level	Kim et al., 2009
miR172	*Zea mays*	Delay	Post-transcriptional level	Wu et al., 2016
miR319	*Arabidopsis thaliana*	Promote	Post-transcriptional level	Schommer et al., 2008
miR840	*Arabidopsis thaliana*	Delay	Post-transcriptional level	Yujun et al., 2019
ONAC054	*Oryza sativa*	Promote	Post-transcriptional level	Sakuraba et al., 2020
PtRD26	*Populus*	Delay	Post-transcriptional level	Wang et al., 2021
SlymiR208	*Solanum lycopersicon*	Promote	Post-transcriptional level	Zhang Y. et al., 2020
ScMYB2	*Saccharum officinarum*	Promote	Post-transcriptional level	Guo X. et al., 2017
u11-48k	*Arabidopsis thaliana*	Promote	Post-transcriptional level	Xu et al., 2016
eIF5A	*Picrorhiza kurrooa*	Delay	Translation level	Parkash et al., 2014
LreEF1A4	*Petunia hybrida*	Delay	Translation level	Sun et al., 2020
SPL33	*Oryza sativa*	Delay	Translation level	Wang et al., 2017
AtSARK	*Arabidopsis thaliana*	Promote	Post-translational level	Xu et al., 2011
AtWAKL10	*Arabidopsis thaliana*	Delay	Post-translational level	Li L. et al., 2021
ATG4a/4b	*Arabidopsis thaliana*	Delay	Post-translational level	Yoshimoto et al., 2004
ATG9	*Arabidopsis thaliana*	Delay	Post-translational level	Hanaoka et al., 2002
ATG10	*Arabidopsis thaliana*	Delay	Post-translational level	Phillips et al., 2008
ATG18a	*Arabidopsis thaliana*	Delay	Post-translational level	Xiong et al., 2005
EDR1	*Arabidopsis thaliana*	Delay	Post-translational level	Frye et al., 2001
MKK4/5,MPK1/2	*Arabidopsis thaliana*	Promote	Post-translational level	Zhang Y. et al., 2020
MAPKKK18	*Arabidopsis thaliana*	Promote	Post-translational level	Matsuoka et al., 2015
MPK6/MKK9	*Arabidopsis thaliana*	Promote	Post-translational level	Zhou et al., 2009
GmSARK	*Glycine max*	Promote	Post-translational level	Li et al., 2006
OsMAPKKK1	*Oryza sativa*	Promote	Post-translational level	Wang et al., 2015
PUB12/PUB13	*Arabidopsis thaliana*	Delay	Post-translational level	Zhou et al., 2015
RPN10	*Arabidopsis thaliana*	Promote	Post-translational level	Lin et al., 2011
RPN5a	*Arabidopsis thaliana*	Promote	Post-translational level	Book et al., 2009
SAUR49	*Arabidopsis thaliana*	Promote	Post-translational level	Wen et al., 2020
SERK4	*Arabidopsis thaliana*	Delay	Post-translational level	Li et al., 2019
UPL5	*Arabidopsis thaliana*	Delay	Post-translational level	Zentgraf et al., 2010
UBP12/UBP13	*Arabidopsis thaliana*	Promote	Post-translational level	Park et al., 2019
UBA2	*Arabidopsis thaliana*	Promote	Post-transcriptional level	Kim et al., 2008

### Chromatin Level

In eukaryotic cells, DNA is packaged into chromatin and its functional units are nucleosomes. The basic unit of chromatin is the nucleosome core particle, a structure in which ∼146 bp of DNA is wrapped around a protein octamer consisting of two subunits each of core histones H2A, H2B, H3, and H4 (Luger et al., 1997; Davey et al., 2002; Marino-Ramirez et al., 2005). The globular region of the histone forms the core of the nucleosome, while the N-terminal tail protrudes from the nucleosomes and is enriched for various post-translational modifications (PTMs), including acetylation, methylation, phosphorylation, and ubiquitination (Bannister and Kouzarides, 2011). These modifications have important regulatory roles, including gene repression, gene activation, and replication (Kouzarides, 2007; Morgan and Shilatifard, 2020). Histone modifications and the enzymes that implement them can facilitate chromatin compaction, nucleosome dynamics, and transcription. These modifications can respond to intrinsic and external stimuli (Kouzarides, 2007). Dysregulation of these processes can alter the balance of gene expression and thus is often observed in many human diseases or plant development, either by gain or loss of function, overexpression, repression through promoter hypermethylation, chromosomal translocation, or mutation of histone-modifying enzymes/complexes, or even histone modification sites (Zhao and Shilatifard, 2019).

Previous investigations revealed that epigenetic modification participates in the plant leaf senescence process. Chromatin immunoprecipitation sequencing (ChIP-seq) analysis using the trimethylation of histone H3 at lysine 4 (H3K4me3) and the trimethylation of histone H3 at lysine 27 (H3K27me3) antibodies reveals the relationship between histone modifications and leaf senescence in *Arabidopsis* (Brusslan et al., 2012, 2015). Mutation of histone deacetylase *AtHD1*, a histone modification-related gene, altered leaf senescence process in *Arabidopsis* (Pandey et al., 2002). The histone acetylation status of specific parts of chromatin is determined by histone acetylases (HATs) and histone deacetylases (HDACs) and their relative activities. Histone acetyltransferase 1 (HAC1) promotes leaf senescence by targeting Ethylene-responsive transcription factor ERF022, a positive regulator of leaf senescence (Hinckley et al., 2019). HISTONE DEACETYLASE 9 (HDA9), HDA15, HDA19, HISTONE DEACETYLASE 2C (HD2C), and SIRTUIN 1 (SRT1) play a potential role in promoting leaf senescence (Buszewicz et al., 2016; Zheng et al., 2016; Liu et al., 2017; Ueda et al., 2018; Hu et al., 2019; Shen et al., 2019). ChIP-seq and fluorescence *in situ* hybridization (FISH) reveal that the chromatin structure changes as the leaf ages in *Arabidopsis*. Overexpression of *SUVH2*, a SU(VAR)3-9 (KMTase1) histone methyltransferase gene, delayed leaf senescence by increasing the dimethylation of histone H3 at lysine 27 (H3K27me2) and H3K27me3 levels in the promoter region of *WRKY53*, a master positive regulator of leaf senescence, and then suppressing its transcripts (Ay et al., 2009). JUMONJI DOMAIN-CONTAINING PROTEIN 16 (JMJ16), a specific H3K4 demethylase containing JmjC-domain, regulates negatively leaf senescence through repressing the expression of *WRKY53* and *SENESCENCE-ASSOCIATED GENE 201* (*SAG201)*, two positive regulators of leaf senescence in *Arabidopsis*. Moreover, genome-wide analysis reveals widespread hypermethylation of H3K4me3 at JMJ16 binding genes, including *WRKY53* and *SAG201*, and coordinated upregulation of their expression in the *jmj16* mutant compared with the wild type (Liu et al., 2019). To screen the upstream regulator of NONYELLOWING1 (NYE1) that regulates chlorophyll degradation during leaf senescence by Yeast one-Hybrid (Y1H) approach, the histone H3K27me3 demethylase RELATIVE OF EARLY FLOWERING6 (REF6) was found to directly interact with the NYE1/2 promoter through its zinc finger domain and up-regulates gene expression of positive regulators of leaf senescence such as *ETHYLENE INSENSITIVE 2* (*EIN2*) and *ORE1* (Wang et al., 2019). In addition, overexpression of *SUVH2* also inhibits the gene expressions of senescence-associated WRKY (Sen-WRKY) and Sen-NAC (NAM/ATAF/CUC) transcription factors, central components of the leaf senescence process, in *Arabidopsis* leaves upon treatment with bleomycin (BLM), a genotoxic chemical that induces double-strand breaks (DSBs) (Li et al., 2020a).

ATP-dependent chromatin remodeling enzyme involved in chromatin remodeling is also associated with leaf senescence. Mutations of *DEFECTIVE IN RNA-DIRECTED DNA METHYLATION 1* (*DRD1*) and *DECREASED DNA METHYLATION 1* (*DDM1*), two SWI2/SNF2 chromatin remodeling proteins, delay leaf senescence (Cho et al., 2016). In contrast, loss-of-function of *BRAHMA* (*BRM*), another SWI/SNF2 chromatin remodeling ATPase (Archacki et al., 2017), accelerates leaf senescence (Efroni et al., 2013; Li C. et al., 2016). Further studies are needed in the future to reveal how various epigenetic modifications coordinately regulate leaf senescence.

### Transcription Level

Large-scale reprogramming of the transcriptome is a core step in plant leaf senescence. Approximately a dozen percent of genes are up-regulated or down-regulated during leaf senescence in *Arabidopsis*. Furthermore, master TFs-mediated transcriptional regulation plays a crucial role in the regulation of leaf senescence (Zentgraf et al., 2010; Breeze et al., 2011). A WRKY transcription factor is one of the plant-specific TF families controlling the leaf senescence process. Members of WRKY TFs, including WRKY6, WRKY22, WRKY42, WRKY45, WRKY46, WRKY53, WRKY54, WRKY55, WRKY57, WRKY70, and WRKY75, coordinate with endogenous hormones to finely regulate the leaf senescence process (Robatzek and Somssich, 2001; Miao et al., 2004; Zentgraf et al., 2010; Zhou et al., 2011; Besseau et al., 2012; Jiang et al., 2014; Chen et al., 2017; Guo P. et al., 2017; Niu et al., 2020; Wang et al., 2020). Recently, WRKY46 was found to interact with Non-expressor of Pathogenesis-Related gene 1 (NPR1) and combined with the WRKY6 promoter to induce its expression in response to SA signals, thereby establishing an NPR1-WRKY46-WRKY6 signaling cascade to regulate leaf senescence (Zhang D. et al., 2021). Several WRKY TFs coordinate leaf growth and senescence in plants, including GhWRKY42 and GhWRKY91 in *Gossypium hirsutum*, BrWRKY6 in cabbage (Brassica rapa), CpWRKY71 in *Chimonanthus praecox*, OsWRKY93 in *Oryza sativa*, BnaWSR1 (WRKY regulating SA and ROS 1) and BnaWGR1 (WRKY generating ROS 1) in *Brassica napus* (Fan et al., 2018; Gu et al., 2018, 2019; Yang et al., 2018; Huang et al., 2019; Cui et al., 2020; Li Y. et al., 2021).

NAC family is one of the largest gene families in plants and plays a central role in regulating leaf senescence. NAC TFs function as positive regulators of leaf senescence, including ORE1/ANAC092, AtNAP/ANAC029, ORE1 SISTER1 (ORS1/ANAC059), ATAF2/ANAC081, ATAF1/ANAC002, ANAC019, AtNAC3/ANAC055, RESPONSIVE TO DESICCATION 26 (ATRD26/ANAC072), ANAC102, ANAC032, ANAC046, ANAC016, and NAC TRANSCRIPTION FACTOR-LIKE 9 (NTL9) or negative regulators such as JUNGBRUNNEN1 (JUB1/ANAC042), ANAC017, VND-INTERACTING1 (VNI1/ANAC082), VND-INTERACTING2 (VNI2/ANAC083), ANAC090, and ANAC075 (Guo and Gan, 2006; Yoon et al., 2008; Kim et al., 2009, 2013; Balazadeh et al., 2011; Nakashima et al., 2012; Wu et al., 2012; Hickman et al., 2013; Garapati et al., 2015; Lee et al., 2015; Takasaki et al., 2015; Kim H. J. et al., 2016, 2018; Li S. et al., 2016; Mahmood et al., 2016; Oda-Yamamizo et al., 2016; Woo et al., 2016; Nagahage et al., 2020; Kan et al., 2021). Future studies need to investigate whether there is communication between these positive and negative regulatory NAC-TFs, which will help to gain insight into the fine regulatory mechanisms of leaf senescence. On the one hand, to investigate whether there are direct interactions between these factors and whether they are synergistic or antagonistic to each other. On the other hand, ChIP-Seq data should be used to analyze whether their target genes overlap to develop a gene regulatory network of leaf senescence. Indeed, some studies have already started to address this aspect. For example, members of NAC-TFs and WRKY-TFs have been found to interact with each other to change the expression of downstream target genes, which in turn triggers leaf senescence (Kim et al., 2009; Zentgraf et al., 2010; Balazadeh et al., 2011; Besseau et al., 2012; Kim H. J. et al., 2016, 2018). Since the process of leaf senescence is accompanied by nutrient return, genes that regulate senescence are likely to regulate crop yield. In supporting this hypothesis, several NAC-TFs regulate crop yield by fine-tuning the initiation and progression of leaf senescence, such as NAM-B1 and TaNAC-S in wheat (Uauy et al., 2006; Zhao et al., 2015; Kang et al., 2019; Sakuraba et al., 2020; Yang et al., 2020; Yan et al., 2021), which provides a molecular strategy to improve crop yield or quality by finely regulating the leaf senescence process.

The basic helix-loop-helix (bHLH) family TFs also regulate leaf senescence. Members of bHLH subgroup IIIe factors, including myelocytomatosis protein 2 (MYC2), MYC3 and MYC4, antagonistically interact with the bHLH subgroup IIId factors bHLH03 (JAM3), bHLH13 (JAM2), bHLH14, and bHLH17 (JAM1), and mediate JA-induced leaf senescence by directly binding the promoter of *SAG29* in Arabidopsis (Qi et al., 2015; Goossens et al., 2017). In addition, MYC5 also positively regulates JA-induced leaf senescence (Song et al., 2017). Darkness induces the protein accumulation of JASMONATE ZIM-domain 7(JAZ7), which in turn inhibits dark-induced leaf senescence by suppressing MYC2 (Yu et al., 2016). In rice, OsMYC2 acts as a positive regulator of leaf senescence by regulating the transcript levels of *SAG*s (Uji et al., 2017). These findings suggest that MYC2 regulates leaf senescence via multiple signaling pathways. ACTIVATION-TAGGED BRI1 (BRASSINOSTEROID-INSENSITIVE1)-SUPPRESSOR1 (ATBS1)-INTERACTING FACTOR2 (AIF2) is a non-DNA-binding bHLH TF and delays dark or BR-induced leaf senescence (Kim et al., 2020). Phytochrome-interacting bHLH transcription factors (PIFs) such as PIF4 and PIF5 promote leaf senescence under natural or dark conditions in Arabidopsis (Sakuraba et al., 2014; Song et al., 2014; Li N. et al., 2021). MdbHLH3 regulates leaf senescence by promoting the expression of *dehydratase-enolase-phosphatase complex 1* (*MdDEP1*) in *Malus Domestica* (Hu et al., 2020). These results imply that bHLH TFs are involved in the regulation of leaf senescence in both annuals and perennial woody plants.

There is growing evidence that multiple TF families of genes are involved in the regulation of leaf senescence, including MYB-TFs such as MYB2 (Buchanan-Wollaston et al., 2005), MYB DOMAIN PROTEIN R1 (MYBR1) (Jaradat et al., 2013), MYB HYPOCOTYL ELONGATION-RELATED (MYBH) (Huang et al., 2015), AtMYBL (Zhang et al., 2011), OsMYB102 (Piao et al., 2019), ScMYB2S1 (Guo J. et al., 2017); PLANT A/T-RICH SEQUENCE- AND ZINC-BINDING PROTEIN (PLATZ) family transcription factor (ORE15) (Kim J. H. et al., 2018); AP2/ERF transcription factors such as CRF1/2/3/5/6 in Arabidopsis (Raines et al., 2016), SlERF36 in Tomato (Upadhyay et al., 2013), and SUBMERGENCE1A (SUB1A) in Rice (Fukao et al., 2012); AP2/DREB transcription factors (DEAR1 and Rap2.4f) (Tsutsui et al., 2009; Xu et al., 2010); CCCH zinc-finger family [K-homolog (KH) proteins, KHZ1 and KHZ2] (Yan et al., 2017); AUXIN RESISTANT 3 (AXR3)/INDOLE-3-ACETIC ACID INDUCIBLE 17 (IAA17), one member of Auxin response factors (ARF) family, is a positive regulator of natural leaf senescence (Shi et al., 2015); TEOSINTE BRANCHED1/CYCLOIDEA/PCF (TCP) family transcription factor (TCP2/4/10) (Schommer et al., 2008); Homeodomain-leucine zipper family (REVOLUTA) (Xie Y. et al., 2014); MADS box transcription factors such as FOREVER YOUNG FLOWER (FYF) in Arabidopsis and SlFYFL in Tomato (Chen et al., 2011; Xie Q. et al., 2014; Guo X. et al., 2017), as well as RAV family transcription factor (RAV1) (Woo et al., 2010).

A large number of studies have shown that TF plays a key regulatory role in leaf senescence, however, most of the studies have mainly focused on a few families, including the NAC or WRKY families, and more studies are needed in the future to analyze whether other family TFs are also involved in leaf senescence. A recent transcriptomic study revealed that 115 Sen-TFs from 31 families are involved in autumn leaf senescence in poplar (Wang et al., 2021), further supporting this suggestion.

### Post-transcriptional Level

Post-transcriptional regulation, including RNA editing, polyadenylation, mRNA stability, and alternative splicing, is related to leaf senescence. Multiple organellar RNA editing factors 9 (MORF9), one of the core proteins of plant editosomes, are involved in the RNA editing in chloroplasts, and its mRNA level declined in senescent leaves (Tian et al., 2019). MicroRNA (miRNA) is involved in leaf senescence by regulating the expression of *SAG* genes. For example, miR156, miR164, miR172, and miR840 regulate leaf senescence by suppressing their target genes (Kim et al., 2009; Wu et al., 2016, 2020; Tian et al., 2019; Yujun et al., 2019; Roussin-Leveillee et al., 2020). In addition to miRNAs, circular RNA (circRNA), and long non-coding RNA (lncRNAs) participate in leaf senescence of rice by a competitive endogenous RNA (CeRNA) network (Huang et al., 2021a, b). Interestingly, EIN3 and clock-associated PSEUDO-RESPONSE REGULATOR 9 (PRR9) up-regulate the transcription level of *ORE1* by inhibiting the transcription of *miR164* (Li et al., 2013; Kim H. et al., 2018). miR398 participates in regulating leaf senescence by post-transcriptional regulation of *ASCORBATE PEROXIDASE 6* (*APX6*) (Chen et al., 2021). *SlymiR208* regulates leaf senescence by controlling the expression of isopentenyl transferases *SlIPT2* and *SlIPT4* (Zhang Y. et al., 2020).

Alternative splicing (AS) is widely used in RNA splicing and processing after gene transcription in higher eukaryotes, which can increase the diversity of transcriptome and proteome. AS events can be mainly classified into five categories: IR, skipping exon and mutually exclusive exons, as well as alternative 5’-splice sites and alternative 3’-splice sites. In animals, splicing factors control cellular senescence by regulating the splicing process of RNA precursors (Fregoso et al., 2013). In plants, AS acts as a regulatory mechanism of plant development or adaptation to environmental stress factors. RNA splicing factor RNA-BINDING PROTEIN 25 (RBM25) responds to ABA stress in *Arabidopsis* (Zhan et al., 2015). Loss-of-function of *CRM FAMILY MEMBER SUBFAMILY 4* (*CFM4*) leads to abnormal rRNA processing during chloroplast RNA splicing, and exhibited plant growth retardation and delayed senescence (Lee et al., 2014). An interesting discovery shows that the differential expression of sugarcane MYB TF ScMYB2 alternative splicing transcripts may be an important post-transcriptional regulatory mechanism for controlling drought stress and leaf senescence (Guo J. et al., 2017).

The splicing mechanism occurs in the spliceosome, which is composed of five small nuclear RNAs (snRNAs) and a series of related protein factors. The spliceosome can recognize the splice site of the precursor RNA and catalyze the splicing reaction. There are major spliceosomes (U2) and minor spliceosomes (U12) that support splicing functions (Sharp, 2005). Although the splicing efficiency of the U12-type spliceosome is relatively lower, splicing errors will affect the normal growth and development of plants. The U12-type intron-specific small spliceosome mainly removes the small U12 intron from the precursor mRNA. Mutation of *u11-48k* causes defects in growth and development, such as short plant size, increased lotus-like leaves, and delayed senescence (Xu et al., 2016), indicating that the regulation of the RNA splicing process has a potentially important effect on plant leaf senescence. ETHYLENE RESPONSE FACTOR4 (ERF4) has two different isoforms, ERF4-R and ERF4-A, produced by alternative polyadenylation of its pre-mRNA. ERF4-R, contains an ERF-associated amphiphilic repression (EAR) motif and acts as a repressor, whereas the other form, ERF4-A, is lacking this motif and acts as an activator. *ERF4-R* and *ERF4-A* can directly bind to the promoter of *CATALASE3* (*CAT3*) but have antagonistic effects on gene expression. The ratio of *ERF4-A* to *ERF4-R* mRNA changed as the plant ages and caused a complex age-dependent regulation of CAT3 activity. Interestingly, overexpression of *ERF4-R* but not of *ERF4-A* led to accelerated senescence (Koyama et al., 2013; Riester et al., 2019). ONAC054 was shown to participate in ABA-induced leaf senescence by directly activating *OsABI5* in rice (Sakuraba et al., 2020). Interestingly, the *ONAC054* transcript (*ONAC054*α) has an alternatively spliced form, *ONAC054*β, encoding a small truncated protein. Overexpression of *ONAC054*α or *ONAC054*β promotes leaf senescence (Sakuraba et al., 2020). A recent study reported that an alternative splicing event retaining the first intron of the *PtRD26* pre-mRNA occurred in a senescence-associated manner in poplar. The intron retention (IR) event in *PtRD26* led to an alternative splicing variant, PtRD26^IR^, which encodes a truncated protein. PtRD26^IR^ forms heterodimers with multiple hub Sen-NAC TFs, including PtNAC039, PtNAC055, PtNAC076, PtNAC086, PtNAC099, and PtNAC109, represses their DNA binding activity to target genes, and delays age-, dark,- and PtRD26-induced leaf senescence in poplar, tobacco, and *Arabidopsis*. PtRD26 regulates Sen-NAC TFs by directly binding their promoters or indirectly through protein-protein interactions using its splicing variant, PtRD26^IR^, thereby forming a multiply-interlocked feed-forward loop to finely tune the leaf senescence process. Functional analysis of senescence-associated splicing factors (SF) revealed that *PtU2A2A*, *PtU2A2B-1*, or *PtU2A2B-2* (U2 auxiliary factor large subunit A or B) are involved in AS of PtRD26^IR^. Silencing separately or simultaneously of these SFs significantly decreased the transcript levels of *PtRD26^IR^* and accelerated leaf senescence. Based on these findings, it is found that the products of AS have different functions and regulate plant development such as plant senescence through different mechanisms. With the application of multi-omics technology, more AS events will be found to be involved in the regulation of leaf senescence, which will Further deepen the mechanistic understanding of plant aging.

### Translation Level

Senescence is a long-term state of cell cycle arrest arising from cells that have suffered sublethal damage. Although senescent cells no longer replicate, they remain metabolically active and further develop a distinct and stable phenotype not seen in proliferating cells (Guo and Gan, 2005; Lim et al., 2007). On the one hand, along with leaf senescence, a large number of proteins are degraded and translation efficiency decreases; on the other hand, senescence-specific regulatory factors are synthesized to inhibit or retard the leaf senescence process (Guo and Gan, 2005; Lim et al., 2007). Thus, translation in senescent cells paradoxically includes a general inhibition of translation triggered by numerous stresses and a selective increase in translation of specific proteins, including SAG protein.

Mutation of *ORE4*, which encodes the plastid ribosomal small subunit protein 17 that is a component of the plastid ribosome, reduces the translation rate in the chloroplast and thus extends leaf longevity in *Arabidopsis* (Woo et al., 2002), suggesting a possible link between decreased metabolism and extended longevity of the leaves. Translation initiation, the first step in the protein synthesis process, is the main regulatory step controlling translation and involves a large number of translation initiation factors. Studies in plants have revealed that these translation initiation factors affect various aspects of plant growth and development, in addition to their role in protein synthesis (Wang et al., 2001). Mutation of *EUKARYOTIC ELONGATION FACTOR 5A* (*eIF5A*) significantly inhibits plant nutrition and reproductive growth and delays leaf senescence in *Arabidopsis* and Picrorhiza (*Picrorhiza kurrooa Royle ex Benth.*) (Reviron et al., 1992; Parkash et al., 2014). Translation initiation factor eIF3h is involved in the signal activation and restart of rapamycin (TOR) and affects the growth and development of plants (Schepetilnikov et al., 2013). In addition, eukaryotic translation elongation factors (eEF) also involved leaf senescence. For instance, mutation of *Spotted Leaf 33* (*spl33*), encoding a eEF1 alpha (eEF1A)-like protein, induces early leaf senescence (Wang et al., 2017). Ectopic expression of *Lilium regales Eukaryotic translation elongation factor 1 alpha 4* (*LreEF1A4*), encoding the α subunit of elongation factor 1 from a *Lilium regale* cucumber mosaic virus (CMV), delayed leaf and flower senescence in petunia (*Petunia hybrida*) (Sun et al., 2020). Interestingly, two subunits of ribulose 1,5-bisphosphate carboxylase/oxygenase (Rubisco), the key enzyme that determines the rate of carbon assimilation in photosynthesis, is controllable at the translation level, and affect plant growth and development, including the leaf senescence process (Suzuki and Makino, 2013; Woo et al., 2013).

### Post-translational Level

Post-translational modifications (PTM), including methylation, acetylation, phosphorylation, ubiquitination, and deubiquitination affect the structure and function of proteins. Previous studies found that the PTM of a large number of SAG proteins changed with leaf senescence (Wang and Schippers, 2019). This implies a close relationship between leaf senescence and PTM, but the causal relationship is not clear.

Transcriptomics analysis reveals that a large number of SAGs are involved in PTM, such as receptor-like kinase (RLK) and mitogen-activated protein kinase (MAPK) (Ahmad and Guo, 2019). RLK is an ideal candidate for senescence-inducing signal receptors, which often have an N-terminal extracellular binding domain for ligand binding, a transmembrane domain spanning the plasma membrane, and a cytoplasmic kinase domain (Shiu and Bleecker, 2001; Gish and Clark, 2011). The largest subfamily of RLK is the leucine-rich repeat receptor-like protein kinase (LRR-RLK), containing more than 200 members, and lots of them are involved in the regulation of leaf senescence (Shiu et al., 2004). GmSARK (*Glycine max* Senescence-Associated Receptor-like Kinase), a senescence-associated LRR-RLK isolated from soybean (*Glycine max*) and its homolog AtSARK in *Arabidopsis* are positive regulators of leaf senescence (Li et al., 2006; Xu et al., 2011). SARK-mediated signaling pathway positively regulates leaf senescence through suppressing SMALL AUXIN-UP RNA 49 (SAUR49), a negative regulator of leaf senescence, and activating SENESCENCE-SUPPRESSED PROTEIN PHOSPHATASE (SSPP), an accelerator of leaf senescence (Xiao et al., 2015; Wen et al., 2020). In contrast, the somatic embryogenesis receptor-like kinase 4 (SERK4) and the cell wall-associated kinase 10 (AtWAKL10) act as the negative regulators of leaf senescence (Li et al., 2019; Li L. et al., 2021). Interestingly, a common receptor can work with multiple receptors in different signaling pathways. AtSARK and SERK4 may be part of the receptor complex that regulates plant aging by acting with other LRR-RLKs (Brandt and Hothorn, 2016; Cui et al., 2018).

The mitogen-activated protein kinase cascade MAPKKK-MAPKK-MAPK is one of the most important signal transduction pathways in plants and animals. Recently, MAP KINASE 4/5 (MKK4/5)-MITOGEN-ACTIVATED PROTEIN KINASE 1/2 (MPK1/2), MITOGEN-ACTIVATED PROTEIN KINASE KINASE KINASE 18 (MAPKKK18), and OsMAPKKK1 have been found to be the positive regulators of leaf senescence (Matsuoka et al., 2015; Wang et al., 2015; Zhang J. et al., 2020). By contrast, Enhanced Disease Resistance 1 (EDR1), a MAPKK, functions as a negative regulator by coordinating biotic stress response and ethylene-induced senescence (Frye et al., 2001; Tang and Innes, 2002). MKK9 phosphorylates the target MPK6, which stabilizes the leaf senescence transcription factor EIN3 by promoting the cleavage and nuclear translocation of ORE3/EIN2 (Zhou et al., 2009; Zhang Y. et al., 2016). These findings suggest that RLKs and MAPKs regulate leaf senescence by affecting the phosphorylation status of target proteins.

The leaf senescence process is accompanied by protein degradation. The main protein degradation pathways are autophagy and the ubiquitin-proteasome system (UPS), which precisely regulate the turnover of organelles and the degradation of abnormal proteins and maintain protein homeostasis. Autophagy and protein ubiquitination are synergistic in the cell. Ubiquitination acts as a signal to induce organelles to target autophagy. Mitophagy and chloroplast protein degradation is the result of the synergistic effect of ubiquitination and autophagy (Geisler et al., 2010; Kikuchi et al., 2020). Interestingly, autophagy seems to prevent aging, whereas the proteasome acts as a positive regulator of aging (Wang and Schippers, 2019). Chaperone-mediated autophagy is one of the main types of autophagy in cells, with high selectivity. Autophagy-related genes (ATG) involved in autophagy are up-regulated with the occurrence of plant senescence (Masclaux-Daubresse et al., 2014). Mutation of several ATG genes, including ATG4a/4b, ATG9, ATG19, and ATG18a, promotes leaf senescence under nitrogen-starvation conditions (Hanaoka et al., 2002; Yoshimoto et al., 2004; Xiong et al., 2005; Phillips et al., 2008; Wang and Schippers, 2019). Although most studies support the role of autophagy in delaying aging, ATG8 promotes senescence by interacting with the ABNORMAL SHOOT3 (ABS3). This non-autophagic ATG8-ABS3 pathway interacts with the classic autophagy pathway to balance aging and survival (Jia et al., 2019). Therefore, the components of autophagy may have a dual role in the initiation and progression of senescence. 26S proteasome is mainly responsible for degrading ubiquitinated proteins. The recognition of ubiquitinated substrates in the process of ubiquitin/proteasome-mediated proteolysis (UPP) is directly mediated by the proteasome subunits RPN10 (REGULATORY PARTICLE NON-ATPase 10) and RPN13. The loss of the potential UPP ubiquitin receptor *RPN10* significantly delays senescence (Lin et al., 2011), and overexpression of *RPN5a* leads to premature senescence (Book et al., 2009). In contrast to the overall up-regulation of ATG genes, transcript levels of only a small part of the proteasome subunit genes were increased during leaf senescence (Guo and Gan, 2012). In the senescent leaf of rape and barley (*Hordeum vulgare* L.), the proteasome is very active (Poret et al., 2016; Velasco-Arroyo et al., 2016). Interestingly, an application of protease inhibitor delays the onset of senescence symptoms (Pak and van Doorn, 2005). Taken together, these observations imply that autophagy and proteasome seem to have different effects on the onset of senescence, and they coordinately regulate the progression of leaf senescence.

One of the well-characterized PTMs involved in the regulation of leaf senescence is ubiquitination/deubiquitination modification. Protein ubiquitination requires the synergy of ubiquitin activation (E1), ubiquitin-binding (E2), and ubiquitin ligase (E3). Members of E2 and E3 have been found to be involved in the regulation of leaf senescence (Shu and Yang, 2017; Park et al., 2018). Among them, RING-type E3 and U-box-type E3 ligases have been shown to act as regulators of leaf senescence by mediating ABA signaling. For example, PLANT U-box (PUB) E3 ubiquitin ligase PUB12 and PUB13 ubiquitinated FLS2 (FLAGELLIN-SENSITIVE 2) for protein degradation, thereby down-regulating flagellin signaling and negatively regulating stress-induced leaf senescence (Zhou et al., 2015). In addition, HECT-type ubiquitin E3 ligase (UPL1-UPL7) plays a critical role in cell death and leaf senescence (Lan and Miao, 2019). Mutation of *UBIQUITIN PROTEIN LIGASE 5* (*UPL5*) leads to the accumulation of *WRKY53* and induces early leaf senescence (Zentgraf et al., 2010). Ubiquitin-specific protease (UBP1)-associated protein 2a (UBA2a), UBA2b, and UBA2c positive regulators of leaf senescence (Kim et al., 2008). Likewise, the potato (*Solanum tuberosum*) RNA-binding protein StUBA2a/b is homologous to *Arabidopsis* UBA2s. Constitutive overexpression of *StUBA2a/b* increases the expression of the *SAG13* gene, pathogen-related genes (PR), and autophagy-related genes, and promotes leaf senescence in *Arabidopsis* (Na et al., 2015). The process of protein ubiquitination is reversible, and deubiquitinating enzymes (DUBs) can remove mono-ubiquitin molecules or polyubiquitin chains on proteins. UBP is the largest DUB subfamily, and members of the UBP family are involved in a variety of physiological processes, including leaf senescence (Zhou et al., 2017). Out of them, UBIQUITIN-SPECIFIC PROTEASE 12 (UBP12) and UBP13 are involved in the regulation of circadian clock and flowering (Cui et al., 2013), and accelerate nitrogen starvation-induced leaf senescence by counteracting the effect of E3 ligase NLA (Nitrogen Ubiquitin-Protein Ligases DNA) to maintain the homeostasis of ORE1 (Park et al., 2019).

## Conclusions and Perspectives

Leaf senescence is a highly complex process of orderly degradation of cell structure and is controlled by multiple layers regulatory network (Figure 1), in which different regulatory factors at different levels may interact to fine-tune the initiation and progression of leaf senescence (Table 1). Although regulation is artificially divided into multiple levels (Woo et al., 2013), leaf senescence is a highly dynamic regulatory process (Woo et al., 2019), and there is no single way to regulate it. For example, changes in chromatin structure affect gene expression, protein translation, and thus the function of transcription factors, which in turn cause changes in the senescence process of plant leaves. Moreover, the regulation of leaf senescence involves not only the interactions between proteins, proteins, and DNA, but also the exchange of information between cells and organelles, thus synergistically regulating the initiation of leaf senescence, which guarantees the return of nutrients and the survival of plants. Therefore, we should combine genome, transcriptome, proteome, metabolome, and the latest translation comics data to discuss the general mechanism of regulate senescence and understand how senescence and death are systemically integrated within the entire plant (Kim J. et al., 2016).

With the aid of forwarding or reversing genetics strategies and the development of multi-functional CRISPR genome editing technology, a large number of senescence-related mutants will be generated. For example, quintuple mutants of *oss40s-cr* generated using CRISPR technology displays stay-green phenotypes (Habiba et al., 2021), which will further deepen our understanding of leaf senescence. The model plant Arabidopsis has played an important role in revealing the molecular or genetic regulation mechanisms of plant senescence, but we still know little about leaf senescence and do not fully understand the biological significance of senescence (Lim et al., 2007). The relatively short life cycle of Arabidopsis has limitations for our understanding of plant aging. Along with the genomic information revealed for a variety of plants, it provides the possibility to systematically study plant senescence by comparative genomics.

It’s unclear how these transcription factors regulate, such as the WRKY family and NAC family, and epigenetic factors co-regulate the senescence process of plants. The function of hormone signaling on leaf senescence has been widely recognized (Hu et al., 2017). It is necessary to further explore how plant signals and environmental signals are integrated into the hormone signaling pathway, and how post-translational modifications such as phosphorylation and ubiquitination are passed through transcription factors, kinases, and protease, finely control these signals to regulate gene expression and protein turnover during leaf senescence. The senescence symptoms of leaf senescence have always been detected at the organ level. However, in senescent leaves, leaf cells are usually at different developmental ages or senescence stages, which makes it impossible to better understand the biological process of leaf senescence. Fortunately, the application of single-cell sequencing technology may offer the possibility to resolve the cytological basis of leaf senescence.

In addition to the loss- or gain-of-function of mutants, ecotypes of various species will greatly contribute to the understanding of the molecular mechanisms underlying leaf senescence. Through analysis of naturally occurring DNA methylation variation regions (NMRs) between Col-0 and C24 accessions of *Arabidopsis thaliana*, a retrotransposon named NMR19-4 (naturally occurring DNA methylation variation region 19) was identified to be involved in the regulation of leaf senescence (He et al., 2018). NMR19-4 is an environmentally associated epiallele that controls leaf senescence by regulating the expression of PHEOPHYTIN PHEOPHORBIDE HYDROLASE (PPH), which is involved in chlorophyll breakdown (Schelbert et al., 2009; He et al., 2018). By mapping the quantitative trait locus (QTL) of leaf senescence between the Col-0 and Ct-1 accessions of *Arabidopsis thaliana*, ACCELERATED CELL DEATH 6 (ACD6) was identified as the causal gene (Jasinski et al., 2020). Using two rice subspecies indica and japonica, variations were found in the promoter regions of the Stay-Green (OsSGR) gene encoding a chlorophyll-degrading enzyme. This promoter variations trigger higher and earlier induction of OsSGR, which in turn accelerates leaf senescence in indica (Shin et al., 2020).

## Author Contributions

ZL conceived the project and designed the manuscript. HG and XX designed part of the manuscript. Y-MZ collected the data and organized figure. PG organized table. All authors have read and agreed to the published version of the manuscript.

## Conflict of Interest

The authors declare that the research was conducted in the absence of any commercial or financial relationships that could be construed as a potential conflict of interest.

## Publisher’s Note

All claims expressed in this article are solely those of the authors and do not necessarily represent those of their affiliated organizations, or those of the publisher, the editors and the reviewers. Any product that may be evaluated in this article, or claim that may be made by its manufacturer, is not guaranteed or endorsed by the publisher.
